# Autosomal dominant Emery-Dreifuss muscular dystrophy caused by a mutation in the lamin A/C gene identified by exome sequencing: a case report

**DOI:** 10.1186/s12887-022-03662-y

**Published:** 2022-10-17

**Authors:** Kristy Iskandar, Sunartini, Farida Niken Astari, Rizki Amalia Gumilang, Nissya Ilma, Ni Putu Shartyanie, Guritno Adistyawan, Grace Tan, Gunadi, Poh San Lai

**Affiliations:** 1 Neurology Division, Department of Child Health, Faculty of Medicine, Public Health and Nursing, Universitas Gadjah Mada/UGM Academic Hospital, Jl. Kabupaten (Lingkar Utara), Yogyakarta, 55291 Indonesia; 2 Department of Neurology, UGM Academic Hospital, Yogyakarta, Indonesia; 3 Department of Cardiology and Vascular Medicine, Faculty of Medicine, Public Health and Nursing, Universitas Gadjah Mada/ UGM Academic Hospital, Yogyakarta, Indonesia; 4 Faculty of Medicine, Public Health and Nursing, Universitas Gadjah Mada, Yogyakarta, Indonesia; 5 Department of Physical Medicine and Rehabilitation, UGM Academic Hospital, Yogyakarta, Indonesia; 6 Division of Human Genetics, Department of Pediatrics Yong Loo Lin School of Medicine, National University of Singapore, Singapore, Singapore; 7 Pediatric Surgery Division, Department of Surgery/Genetics Working Group/Translational Research Unit, Faculty of Medicine, Public Health and Nursing, Universitas Gadjah Mada/Dr. Sardjito Hospital, Yogyakarta, Indonesia

**Keywords:** Emery-Dreifuss muscular dystrophy, Exome sequencing, Laminopathies, LMNA, Case report

## Abstract

**Background:**

Emery-Dreifuss Muscular Dystrophy (EDMD) is an uncommon genetic disease among the group of muscular dystrophies. EDMD is clinically heterogeneous and resembles other muscular dystrophies. Mutation of the lamin A/C (*LMNA*) gene, which causes EDMD, also causes many other diseases. There is inter and intrafamilial variability in clinical presentations. Precise diagnosis can help in patient surveillance, especially before they present with cardiac problems. Hence, this paper shows how a molecular work-out by next-generation sequencing can help this group of disorders.

**Case presentation:**

A 2-year-10-month-old Javanese boy presented to our clinic with weakness in lower limbs and difficulty climbing stairs. The clinical features of the boy were Gower's sign, waddling gait and high CK level. His father presented with elbow contractures and heels, toe walking and weakness of limbs, pelvic, and peroneus muscles. Exome sequencing on this patient detected a pathogenic variant in the *LMNA* gene (NM_170707: c.C1357T: NP_733821: p.Arg453Trp) that has been reported to cause Autosomal Dominant Emery-Dreifuss muscular dystrophy. Further examination showed total atrioventricular block and atrial fibrillation in the father.

**Conclusion:**

EDMD is a rare disabling muscular disease that poses a diagnostic challenge. Family history work-up and thorough neuromuscular physical examinations are needed. Early diagnosis is essential to recognize orthopaedic and cardiac complications, improving the clinical management and prognosis of the disease. Exome sequencing could successfully determine pathogenic variants to provide a conclusive diagnosis.

## Background

Emery-Dreifuss Muscular Dystrophy (EDMD) (MIM 310,300 and 310,200) is a rare genetic muscular disease with an estimated incidence of 1–9 in 1,000,000 worldwide [1]. It resembles the most common muscular dystrophy, i.e., dystrophinopathy (Duchenne and Becker muscular dystrophy/ DMD and BMD). EDMD has three patterns of inheritance: X-linked recessive, autosomal recessive, and autosomal dominant [2]. Characteristic of EDMD is the presence of contracture in the neck, elbow, and heels in the patient or their relatives [3]. Mutation of the *Lamin A (LMNA)* gene that encodes lamin A/C protein, which causes EDMD, also causes a wide range of other diseases [4]. Thus, the overlapping genotype and phenotype similarities with other muscular dystrophies present diagnostic challenges. A precise diagnosis of EDMD is vital because the disease is associated with life-threatening cardiac conditions. Patients have clinical variabilities in disease progression, life expectancies and prognoses. Hence genetic counselling is essential for affected families once the disease has been diagnosed conclusively.

## Case presentation

**Fig. 1:**
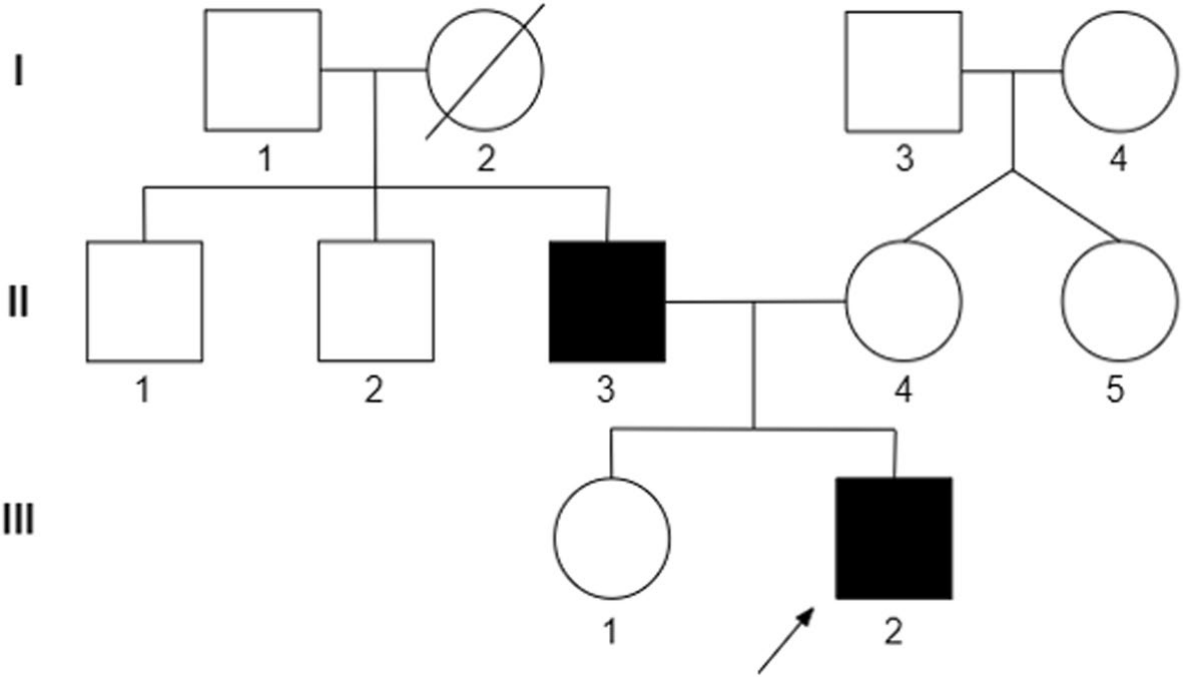
Pedigree of family showing EDMD status of each member. Shaded: affected EDMD

A 2 year-10-month-old male boy of Javanese descent presented to Universitas Gadjah Mada Academic Hospital with weakness of the lower limbs. The boy had an unremarkable birth history from nonconsanguineous parents (Fig. 1). There was no cognitive impairment, no seizure, visual or auditory impairment, and no bowel or bladder dysfunction. He walked at 12 months. Other developmental milestones were also unremarkable. He was vaccinated for age-appropriate immunizations according to the national immunization program guidelines.

**Fig. 2:**
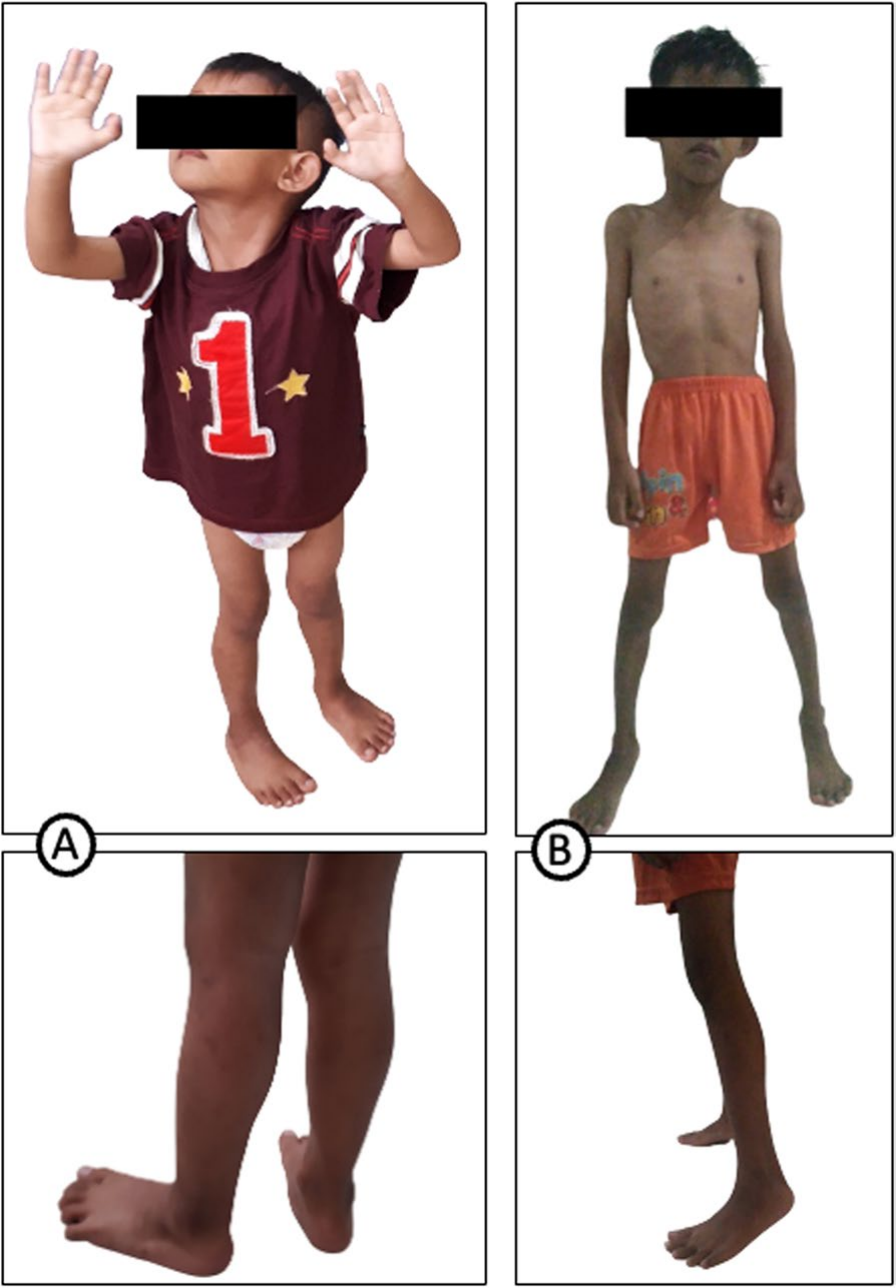
**A** At age 2-year-10-month-old, elbow contractures were not evident, and no profound pseudohypertrophy of the calf and ankle joint contracture in the patient. **B** At age 6-year-old, the patient presented wasting, especially in the upper arm and Achilles contractures

The patient was conscious and afebrile on physical examination with no tachycardia or facial dysmorphic features. There was weakness in the proximal of the upper limbs and distal in the lower limbs. Facial weakness was not detected. No obvious calf pseudohypertrophy and no ankle joint contracture were observed (Fig. 2a). Follow-up at six years old, the patient presented wasting in the upper arm, Achilles contracture, and limitation of neck flexor because of neck contractures (Fig. 2b). Muscle strength examination was 4/5 on the upper and 4/5 on the lower limbs, with no cervical weakness. Swaying movements were present on walking. No toe walking was observed. The sensitivity of all digits was intact. Tendon and cutaneous reflexes were normal. He could walk without support, and the Gower sign was positive. The cardiorespiratory examination was unremarkable. No medical treatment had been given.

Laboratory examinations showed an increased creatine kinase (CK) level of 2485 UI/L, increased LDH level at 1078 U/L, haemoglobin count of 11.6 g/dl, white blood cell count of 9200 cells/μL and normal transaminase level. ECG showed no abnormality of conduction with normal p waves. Echocardiography showed normal structure and function of the heart. Significantly, the patient had no cardiac symptoms at this point.

**Fig. 3:**
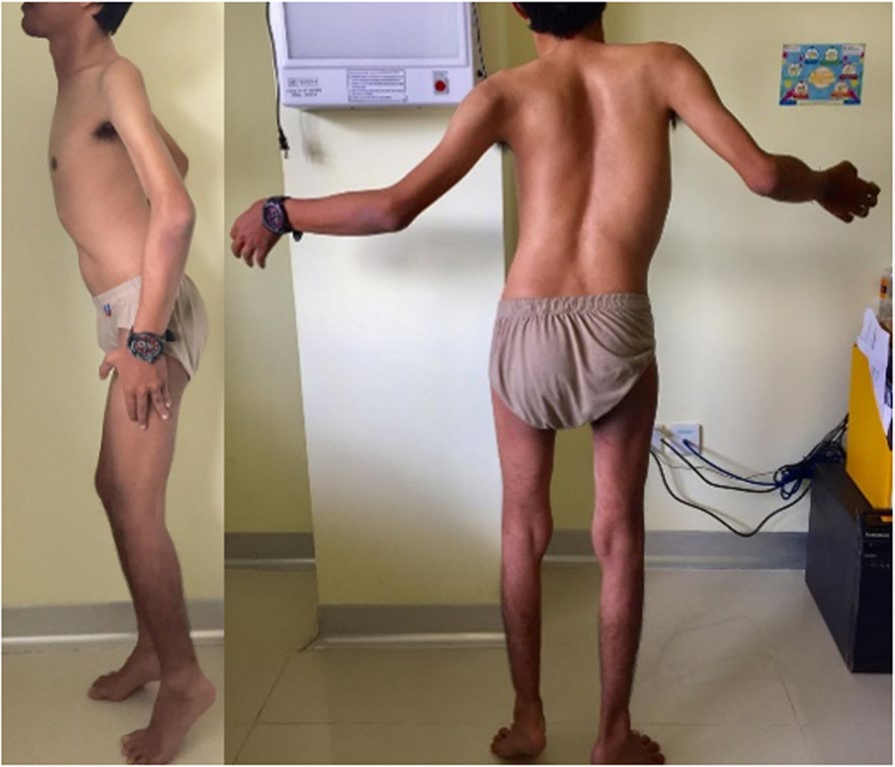
Contractures of the Achilles (toe walking), contractures of the elbow, and atrophy of the scapulohumeral region. The rigidity of the neck was observed in the father

Investigation revealed that was no family history of members except for the father. History taking revealed the father presented with motoric disturbances and was diagnosed with acute flaccid paralysis at eight years old. He started toe walking and could not raise his hands against gravity since he was eight years of age, and there was no history of fever. At the time of the clinical presentation, he was easily exhausted and had difficulty climbing stairs. No shortness of breath and no swelling of the feet were found. On physical examination, there was no facial weakness. He could not bend his neck downwards nor sidewards. He had profound contractures of the elbow and heels (Fig. 3). Muscle wasting and weakness were found in scapulohumeroperoneal regions, with no contractures of fingers. He had decreased physiological reflex and no pathological reflex. Lordosis and scoliosis were detected. He has swaying and tiptoeing in his walk. He could ride a motorcycle by himself and was capable of performing his normal occupation. The father had increased CK level at 518 UI/L, but other blood results were within normal limits. Based on the clinical features, we suspected this was a case of EDMD with a differential diagnosis of limb-girdle muscular dystrophy. No NCS-EMG and MRI findings were available of the patient and the father as they did not consent to these procedures.

**Fig. 4:**
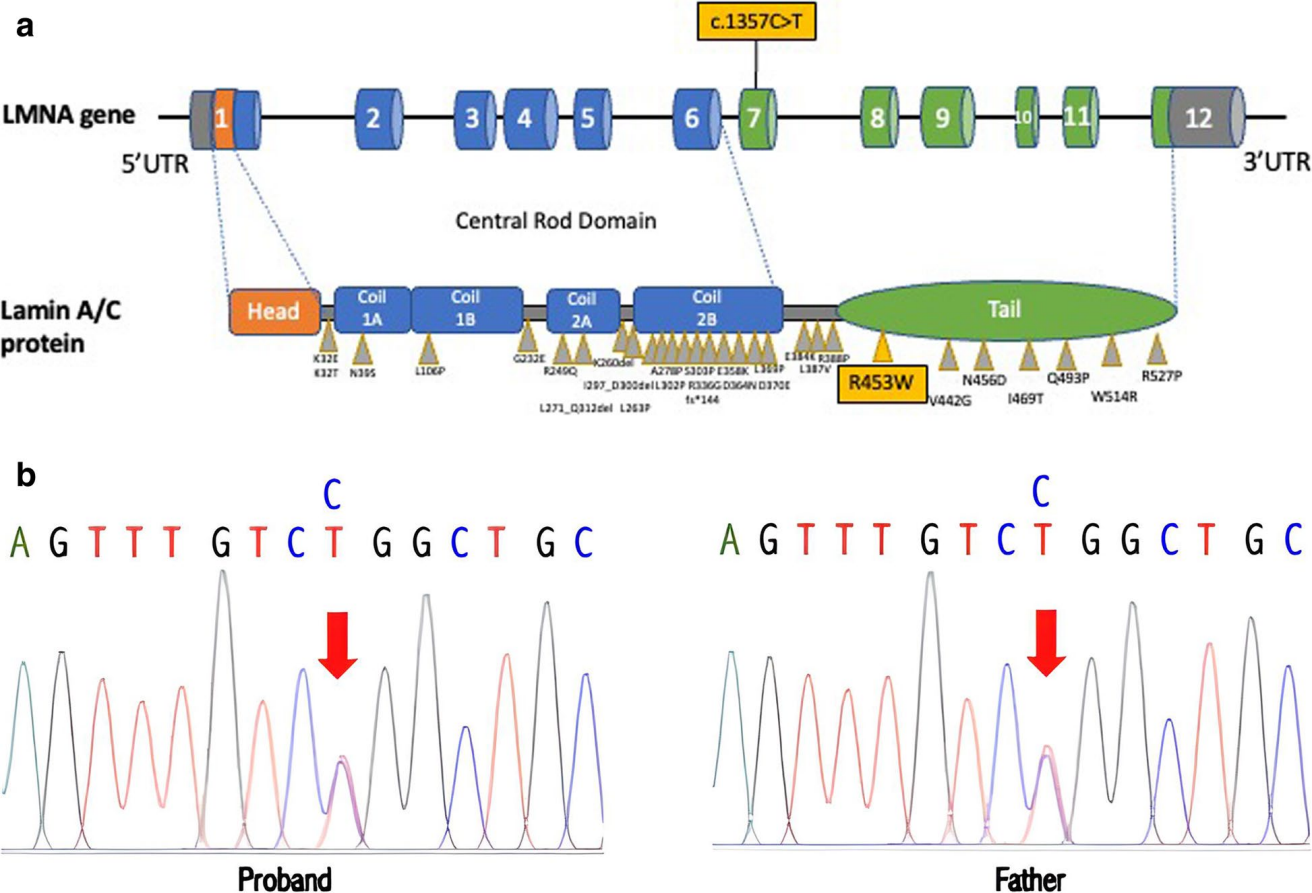
**a** The genetic features reported in EDMD patients. The R453W mutations are located in the tail region, the Ig-like fold of the Lamin A/C proteins. **b** Sanger sequencing confirmation of the son and the father R453W (NM_170707: c.C1357T: NP_733821: p.Arg453Trp) mutation

Exome sequencing was carried out on Illumina Hiseq 4000 platform (Illumina, San Diego, CA) at a mean read depth of 100x. Genomic DNA libraries were prepared by Agilent SureSelect Human All Exon V5 Kit (Agilent Technologies, Santa Clara, CA) following the manufacturer's protocol and sequenced through our laboratory at the National University of Singapore [4]; heterozygous variant involving a C to T transition in exon 7 of the *LMNA* gene (NM_170707: c.C1357T) was found (Fig. 4a). This variant leads to a missense mutation, Arg453Trp (R453W), that was previously reported to cause Autosomal Dominant EDMD. This variant was validated by Sanger sequencing in the patient. Targeted screening of this variant showed presence in the father, confirming this as a familial mutation (Fig. 4b). The variant was also classified as pathogenic based on the ACMG curation guidelines.

**Fig. 5:**
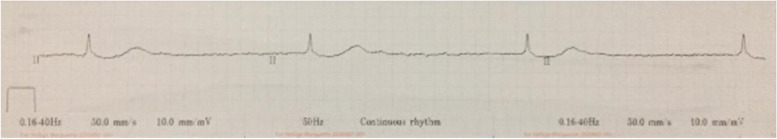
Electrocardiography result showing total atrioventricular block with atrial fibrillation

Knowing the EDMD phenotypes, we further examine the cardiac function of the father. Electrocardiography showed bradycardia with a heart rate of 40–50 bpm and total atrioventricular block with atrial fibrillation (Fig. 5). Echocardiography showed hypokinetic and dilatation of heart muscles, also thrombus suspicion in the left ventricle. Sinus node dysfunction was suspected; thus, atrioventricular block medication was administered. The cardiologist planned for urgent permanent pacemaker placement and further management of cardiomyopathy.

## Discussion and conclusion

Although initially grouped together with the other X-linked muscular dystrophies, most notably the dystrophinopathies forms, EDMD has become accepted as a separate and distinctive type after a thorough clinical evaluation of patients. A triad of presentations characterizes the disease course. The first two characteristic features are the weakness of the proximal muscle in childhood which initially affects the lower extremities, along with elbow flexion contractures and shortening of the Achilles tendon, consequently generating toe walking. Adults with EDMD manifest a waddling gait, lordotic stance and absence of the deep tendon reflexes. The third characteristic consists of arrhythmias, ranging from junctional rhythm (atrial standstill) to atrial fibrillation and even sudden cardiac death [5].

The serum CK level among the affected family members is also increased, although not in the similar range as in DMD or BMD. Other critical differences compared to DMD/BMD are the absence of developmental delay or the calf pseudohypertrophy. In 1966, this disease entity was differentiated and is currently known as X-linked EDMD [6].

EDMD creates a diagnostic challenge due to its similarities in the clinical presentation and laboratory findings with other muscular dystrophies. However, early diagnosis is crucial to prevent early mortality and morbidity from cardiac complications and muscular contractures. It is also essential to provide genetic screening for family members of the patients to determine risk.

In neuromuscular disorder, a nerve conduction study with electromyography is essential to determine which structure is involved. As contracture or spasticity with weakness can be part of an upper motor neuron involvement, an MRI to rule out a central affection or an NCS-EMG with a myopathic pattern to support EDMD may be necessary to support their diagnosis [7]. Moreover, because the father had acute flaccid paralysis since he was eight years old, it is suggested that Charcot Marie Tooth (CMT) should be ruled out by NCS-EMG as some of CMT's variants were thought to be due to *LMNA* gene mutation*.* However, as we did not have data on NCS-EMG and MRI of the father of the case index, molecular analysis was performed to resolve the diagnosis for this case.

EDMD has three main genetic patterns: X-linked recessive, autosomal dominant, and autosomal recessive, with the X-linked EDMD arising from emerin gene chromosome mutation (Xq28) being the most common. Autosomal recessive inheritance is extremely rare [8]. The emerin protein is located in the inner nuclear membrane of body cells, predominantly in skeletal and cardiac muscles. Mutation in the emerin gene causes premature termination in mRNA translation, disrupting protein synthesis and eventually nuclear functioning [9]. The autosomal dominant and recessive patterns of EDMD are known to be caused by mutations of the Lamin A/C genes (*LMNA*) gene on 1q21.2-q21.3. This mutation contributes to the disorder of cardiac and skeletal muscles. Lamin A/C proteins configure the inner nuclear membrane, which plays a significant role in mechanically stabilizing the nuclear envelope and cell signalling. Lamin A/C gene has 12 exons that produce at least four types of RNA via alternate splicing, including lamins A, Aδ10, C and C2. Lamin A and C are intermediate filament proteins. Their defects in the nuclear cells’ mechanical integrity cause disruption in the regulation of tissue-selective transcription alterations and defects in cell proliferation.

From the genetic aspect, two hotspot mutations of *LMNA* have been reported: (1) Arg453Trp/R453W, consistently identified in EDMD, and (2) Arg482Trp/Gln/Leu(R482W/Q/L), consistently identified in patients presenting with partial lipodystrophy (FPLD). Mutations leading to striated muscle laminopathy (EDMD/LGMD1B/DCM-CD) are distributed all along the *LMNA* gene [10] (Fig. 4). As one of the most frequent mutations that are responsible for 16% of AD-EDMD cases, the exchange of arginine 453 by tryptophan (R453W) causes an abnormal nuclear phenotype [11, 12]. Therefore, this mutation is not uncommon. The R453W is a hot spot mutation previously associated with, and some phenotypes reported can be seen in Table 1.

**Table 1:** Phenotype presentations of EDMD that are reported to be associated with R453W

Age (y.o)	Sex	Onset (Age)	Inheri-tance	Phenotype	Cardiac	Ref
8	F	2y	De Novo	Ankle contracture	Sinus arrhythmia	Fan et al. [13]
4	M	2y	De Novo	Ankle contracture	Normal	Fan et al [13]
14	F	4y	De Novo	Ankle contracture, scoliosis	Sinus tachycardia	Fan et al [13]
8	M	2y	De Novo	Elbow & ankle contractures	Normal	Fan et al [13]
54	F	N/A	N/A	Muscle weakness and contractures	Dilated cardiomyopathy, conduction disease	Bernasconi et al. [14]
21	F	N/A	N/A	Muscle weakness and contractures	Dilated cardiomyopathy, conduction disease	Bernasconi et al. [14]
4	M	2y	De Novo	Proximal lower limb weakness, both sides of ankle contractures	Normal	Lin et al. [14]
14	F	4y	De Novo	Proximal and distal lower limb weakness. spine and both sides of ankle contractures	Tachycardia, ultrasonic cardiogram is normal	Lin et al. [15]
3	F	1y	De Novo	LGMD, proximal lower limb weakness, both sides of ankle contractures	Normal	Lin et al. [15]
29	M	4y	De Novo	Weakness in both legs, slow gait, ankle contracture, stiff neck, scoliosis	AV block I	Lee et al. [16]
46	M	N/A	De Novo	Wasting and weakness of the proximal muscles, mild Achilles and elbow contractures, slight spine rigidity, lumbar hyperlordosis	AV block III, AFib, SVT, Hypokinesia of IVS and anterior wall of LV, EF 15%, pacemaker	Madej-Pilarczyk et al. [8]
36	M	N/A	De Novo	Contracture, spine rigidity	SVEB, SVT, VEB, AV Block 1, ICD	Niebroj-Dobosz et al. [16]
44	M	N/A	AD	Contracture, spine rigidity	AV Block 1,2,3, atrial standstill, nsVT, EF 36%, Pacemaker, ICD	Niebroj-Dobosz et al. [17]
21	F	N/A	AD	Contracture, spine rigidity	SVEB, SVT, ICD	Niebroj-Dobosz et al.[17]
34	M	8y	AD	Slowly progressive humero-peroneal muscular weakness. Rigid spine, elbow and Achilles contractures	AV Block III; pacemaker at age 31; died at age 34 of heart failure;	Meinke et al. [18]
37	N/A	N/A	N/A	LGMD, contractures	Afib	Magagnotti et al. [18]
24	M	N/A	AD	Mild proximal weakness. Neck and heel cord contractures	Echocardiogram shows mildly enlarged left atrium and ventricle, mild global hypokinesis and reduced systolic function, estimated LVEF 45%	Scharner et al. [19]
36	M	N/A	N/A	EDMD phenotype	First degree heart block	Scharner et al. [19]
32	M	N/A	De Novo	Limb-girdle, proximal, upper, distal lower muscle. Neck, elbow, ankle, knee contractures	Beta blockers at age 22, pacemaker at age 32	Scharner et al. [19]
19	M	4y	De Novo	Limb-girdle, proximal, upper, distal lower muscle. Neck, elbow, ankle contractures	Normal	Scharner et al. [19]
28	F	6y	AD	Leg weakness (footdrop like gait). Elbow contracture	EMG-diffuse myopathic process; cardiac involvement	Scharner et al. [19]
7	M	3y	De Novo	LGMD, proximal lower extremity weakness, hip contractures	Normal	Scharner et al. [19]
32	N/A	4y	De Novo	No loss of ambulation	N/A	Deconinck et al. [20]
42	N/A	2y	De Novo	Loss of ambulation	N/A	Deconinck et al. [20]
27	N/A	1y	AD	No loss of ambulation	N/A	Deconinck et al. [20]
4	F	2y	AD	Proximal limb muscle weakness, lordosis, calf hypertrophy	Normal	Park et al. [21]
N/A	F	10y	AD	Generalized muscle atrophy, spine rigidity, and spine, elbow, knee, ankle contractures	SVEB, SVT	Niebroj-Dobosz et al. [22]
1 patient	N/A	N/A	N/A	Proximal limb weakness without joint contracture, LGMD 1B	Cardiomyopathy with conduction defect in 34 yo patient	Astejada et al. [23]
5 patients	N/A	N/A	N/A, AD	Joint contractures. 2 patients have rigid spine syndrome. 1 patient has humeroperoneal muscle involvement with Achilles and elbows contractures, and hind neck		Astejada et al. [23]
47	F	N/A	AD	N/A	Afib, ICD	Golzio [24]
49	M	6y	De novo	Elbows and achilles contractures, rigid spine	Afib with appropriately functioning VVIR, Pacemaker	Sanna et al. [25]
16	NA	N/A	N/A	Shoulder, hip, elbow, ankle, rigid spine contractures	None	Vytopil, et al.[26]
10	NA	N/A	AD	Rigid spine	None	Vytopil, et al. [26]
46	NA	N/A	N/A	Rigid spine, axial and proximal myopathies	Conduction defects, pacemaker	Vytopil, et al. [26]
15	F	2y	AD	Proximal weakness. elbow, ankle contractures, mild neck stiff, rigid spine	Normal	Colomer et al. [27]
12	F	2y	AD	Proximal weakness. elbow, ankle contractures, slight neck stiff, rigid spine	Normal	Colomer et al. [27]
46	F	7y	De novo	Proximal weakness. elbow, ankle contractures, stiff neck rigid spine, wheelchair bound 44 years	AV Block 26 y, pacemaker 31 y	Colomer et al. [27]
38	M	Childhood	AD	Limb girdle, upper, proximal, distal, lower muscle weakness. elbow and neck contractures	Pacemaker	Bower et al. [28]
33	M	N/A	AD	Muscle weakness, distal wasting, rigid spine, Achilles contractures and sometimes elbows	Arrythmia	Sewry, et al. [29]
35	M	N/A	AD		Arrythmia	Sewry, et al. [29]
10 months	M	N/A	AD		Normal	Sewry, et al. [29]
17	M	N/A	AD		Ventricular dysfunction	Sewry, et al. [29]
19	N/A	3y	De Novo	Both arms wasting, stiff neck, elbow, hip, Achilles contractures	Arrythmia, ventricular dysfunction	Bonne, et al. [30]
33	N/A	8y	De Novo	Humeroperoneal wasting, elbow, hip, Achilles contractures, stiff neck, rigid spine, kyphosis	Pacemaker	Bonne, et al. [30]
39	M	4y	AD	Mild proximal wasting, elbow, hip, Achilles contractures, stiff neck, rigid spine, scoliosis	Arrhythmia, normal echocardiography	Bonne, et al. [30]
40	M	5y	AD	Proximal wasting, scapular winging, elbow, hip, Achilles contractures, stiff neck, rigid spine, scoliosis	Arrhythmia normal echocardiography	Bonne, et al. [30]
9	M	3y	AD	Scapuloperoneal wasting, elbow, Achilles contractures, stiff neck, rigid spine	Normal	Bonne, et al. [30]
7	F	4y	AD	Scapuloperoneal wasting, elbow, achilles contractures, stiff neck, rigid spine	Normal	Bonne, et al. [30]
49	M	N/A	De Novo	Atypical EMD	Pacemaker, Restrictive cardiomyopathy, died et causa cardiac arrest	Di Barletta et al. [31]
39	M	N/A	X-linked	X-EMD or Rigid Spine Syndrome	Pacemaker	Di Barletta et al. [31]
42	M	N/A	X-linked	X-EMD or Rigid Spine Syndrome	Tachyarrhythmia and AV block	Di Barletta et al. [31]
21	M	N/A	X-linked	X-EMD	None	Di Barletta et al. [31]

AD-EDMD has been reported with a broader clinical spectrum and higher frequency of de novo mutations than the X-linked form. Later and mild involvement of contracture in LGMD1B, which were re-classified as EDMD2, can contribute to its delayed diagnosis [32].

Duchenne muscular dystrophy (DMD) is the most common muscular dystrophy in childhood. For discriminating EDMD from DMD patients, DMD children will have the following features: (a) delay in the acquisition of walking, which usually happens between 16 and 18 months; (b) early pseudohypertrophy of calves; (c) no elbow retractions; (d) CK values up to 100 times the maximum normal value; and (e) increased values of transaminases, which are never observed in patients with EDMD from either emerin or lamin A/C gene defects [33]. Moreover, the neurological examination of the father highlighted both muscular and cardiac characteristics making possible the suspicion of EDMD in our patient. In Indonesia, this is the second case of EDMD reported [14]. Moreover, our case is the first muscular dystrophy report from Indonesia using the whole-exome sequencing approach showing the utility of this approach together with clinical manifestation and usual diagnostic tests for establishing a diagnosis. However, it should be noted that many institutions worldwide do not have access to genetic testing. Although rare, there could be under-reporting of such cases due to a lack of precise molecular diagnosis because of clinical heterogeneity of this disease. This study highlights the importance of comprehensive genetic screening together with clinical features and usual diagnostic tests to further investigate suspicious cases that could resemble some form of DMD.

Most the EDMD cases are sporadic cases. Therefore, presenting the same symptoms in two family members is unusual. Early presentation of EDMD in a boy would be easily mistaken as DMD. EDMD can be inherited by an X-linked pattern, which further shows similarity with DMD. Furthermore, precise diagnosis is essential, as attested in this case, where severe cardiac involvement in the father could be detected and treated earlier.

We report a mutation in *the LMNA* gene underlying an autosomal dominant form of EDMD. EDMD phenotypes resemble the more common form of muscular dystrophy, i.e. dystrophinopathies (DMD/BMD), and may also be inherited in an x-linked inheritance pattern. EDMD should be considered when diagnosing a child with a clinical suspicion of DMD. Early diagnosis, intervention, targeted management, and counselling are crucial to increasing the health and life quality of EDMD patients.

## Data Availability

The datasets used and/or analyzed during the current study are available from the corresponding author on reasonable request.

## Abbreviations

AD: Autosomal Dominant
AV: Atrio Ventricular
AVB: Atrio Ventricular Block
BMD: Becker Muscular Dystrophy
CK: Creatine Kinase
DCM-CD: Familial dilated cardiomyopathy with conduction systems disease
DMD: Duchenne Muscular Dystrophy
ECG: Electrocardiography
EDMD: Emery-Dreifuss Muscular Dystrophy
ICD: Implantable Cardioversion Defibrillator
LGMD: Limb-Girdle Muscular Dystrophy
LGMD1B: Limb-Girdle Muscular Dystrophy Type 1B
LMNA: Lamin A/C
N/A: Not available
PM: Pacemaker
VVIR: Ventricular pacing, ventricular sensing, inhibition response and rate-adaptive, used for ventricular pacemakers
